# Sociodemographic characteristics associated with alcohol consumption and alcohol-related consequences, a latent class analysis of The Norwegian WIRUS screening study

**DOI:** 10.1186/s12889-019-7648-6

**Published:** 2019-10-24

**Authors:** Jens Christoffer Skogen, Tormod Bøe, Mikkel Magnus Thørrisen, Heleen Riper, Randi Wågø Aas

**Affiliations:** 1 Department of Health Promotion, Norwegian Institute of Public Health, Bergen, Norway; 2 Department of Public Health, Faculty of Health Sciences, University of Stavanger, Stavanger, Norway; 3 Alcohol & Drug Research Western Norway, Stavanger University Hospital, Stavanger, Norway; 4 Department of Psychosocial Science, Faculty of Psychology, University of Bergen, Bergen, Norway; 5 Department of Occupational Therapy, Prosthetics and Orthotics, Faculty of Health Sciences, OsloMet – Oslo Metropolitan University, Oslo, Norway; 6 Department of Clinical, Neuro- and Developmental Psychology, Amsterdam Public Health Research Institute, Vrije Universiteit Amsterdam, Amsterdam, the Netherlands

**Keywords:** Alcohol use, Alcohol-related consequences, Socioeconomic status, Alcohol-harm paradox, Latent class analysis

## Abstract

**Background:**

For alcohol, the association with socioeconomic status (SES) is different than for other public health challenges – the associations are complex, and heterogeneous between socioeconomic groups. Specifically, the relationship between alcohol consumption per se and adverse health consequences seems to vary across SES. This observation is called the ‘alcohol harm paradox’. This study aims to describe different patterns of alcohol use and potential problems. Next, the associations between sub-groups characterized by different patterns of alcohol use and potential problems, and age, gender, educational level, full-time employment, occupational level and income is analysed.

**Methods:**

Employing data from the ongoing cross-sectional WIRUS-study, *N* = 4311 participants were included in the present study. Individual response patterns of the ten-item Alcohol Use Disorders Identification Test (AUDIT) were analysed and latent class analysis (LCA) was used to identify latent groups. Next, the associations between the classes identified in the best fitting LCA-model and sociodemographic factors were analysed and presented.

**Results:**

We identified three classes based on the response patterns on AUDIT. Class 1 was characterised by low-level alcohol consumption and very low probability of negative alcohol-related consequences related to their alcohol consumption. Class 2 was characterised by a higher level of consumption, but despite this, class 2 also had a relatively low probability of reporting negative alcohol-related consequences. Class 3, however, was characterised by high levels of alcohol consumption, and a high probability of reporting negative consequences of their consumption. The classes identified were systematically differentially associated with the included measures of SES, with class 3 characterised by younger age, more males and lower educational attainment.

**Conclusions:**

Our findings highlight the interconnectedness of alcohol consumption and alcohol-related consequences. Furthermore, the identified classes and SES yields further insights into to intricate relationship between various socioeconomic factors, alcohol use patterns and related negative consequences.

## Introduction

Alcohol use seems to be unlike other public health challenges when it comes the relationship with socioeconomic status (SES). Lower socioeconomic status is usually related to higher levels of disadvantageous health-related behaviour, such as less exercise, unhealthy diet and smoking [1], resulting in negative health consequences for the individual. For alcohol, the associations are complex, and heterogeneous between socioeconomic groups [2]. Several studies indicate that individuals with higher socioeconomic status consume more alcohol compared to groups in lower social strata [2]. There are exceptions to this pattern, however, and some find this association only among females and in particular countries [3, 4]. In a comparative study of 15 countries, it was for example reported that whereas heavy drinking was related to higher education among women in most countries, it was associated with lower education among men in specific countries, including Norway [4]. Studies also find patterns of more bingeing and higher scores on the Alcohol Use Disorders Identification Test (AUDIT [5, 6];) among men with lower SES [3, 4].

The complexity involved is evident as we observe the risks for negative consequences of alcohol consumption which appear higher among those with lower socioeconomic status compared to those with higher socioeconomic status despite similar consumption levels. This means that the relationship between alcohol consumption per se and adverse health consequences seems to be different across the socioeconomic spectrum, dubbed the ‘alcohol harm paradox’. For each alcohol unit consumed, alcohol-related harm are greater among people with lower socioeconomic status [7]. The mechanisms behind the ‘alcohol harm paradox’ is not very well understood [3, 8]. Broadly, putative mechanisms related to socioeconomic status can be divided into ‘differential exposure’ and ‘differential vulnerability’ [9]; i.e. a situation where different groups have different exposures to a specific factor and a situation where the effect of a given exposure varies between groups, respectively. In relation to alcohol-harm, the most obvious a-priori candidate for differential exposure is the level of alcohol consumption. However, as highlighted in the ‘alcohol harm paradox’, a common finding is that alcohol consumption per se is lower in groups with lower socioeconomic status compared to groups in higher ends of the socioeconomic spectrum, despite higher levels of alcohol-related harm in the former group. For instance, a systematic review and meta-analysis on socioeconomic differences in alcohol-attributable mortality concluded that differences in alcohol exposure does not fully explain the observed association between lower socioeconomic status and alcohol-attributable mortality [10]. Several studies have modified this general finding somewhat, and called for a more detailed investigation into alcohol consumption patterns [8, 11–14]. This may include investigating differential proportions at the extreme ends of the alcohol-consumption spectrum, amount consumed during one drinking session (‘binge drinking’/‘heavy episodic drinking’), density of drinking occasions (e.g. weekend heavy drinking), and type of beverage being consumed, as well as alcohol dependency. As a case in point, although the mean level of consumption in low SES groups is less than for higher SES groups, Lewer and colleagues (2016) found that lower SES groups also were more likely to drink at very high levels compared to other socioeconomic groups [8]. This observation may in part help to explain the paradox. Still, Katikireddi and colleagues (2017) found that low socioeconomic status was consistently associated with raised alcohol-attributable harm (hospital admissions or death) compared to higher socioeconomic status despite controlling for weekly consumption and binge drinking [15]. Other studies have qualified this finding and uncovered specific associations between alcohol-attributable conditions and socioeconomic status, where low - relative to high - socioeconomic status is related to some cancers, stroke, hypertension and liver disease [16]. It has also been suggested that it is not consumption per se that is related to increased morbidity and mortality among those with low socioeconomic status, but the clustering of adverse behaviours such as smoking, being overweight, poor diet and lack of exercise [17]. On the other hand, Böckerman and colleagues using twin data, thus accounting for shared genetic and environmental factors, reported that former drinkers and heavy drinkers had nearly 20% lower earnings compared to moderate drinkers [18]. This finding was robust for controlling for potential confounders, such as pre-existing health and smoking.

In summary, to understand the underpinnings of the paradox there is a need for more studies investigating different aspects of alcohol consumption patterns and consequences of alcohol consumption and the relationship with socioeconomic status. Specifically, there is a need for studies that are able to distinguish groups based on their consumption patterns, the related negative consequences of these patterns and to determine the socioeconomic characteristics that defines those groups. By using a person-centered approach to explore different response patterns not only related to alcohol consumption but also related negative consequences this paper adds to the existing knowledge about what characterises different sub-groups based on their alcohol habits, and how these groups relates to pertinent socioeconomic indicators.

### Aims

The overall aim is to identify and describe sub-groups of alcohol consumption and potential alcohol-related problems and investigate how these sub-groups relate to sociodemographic factors, including indicators of SES. This will be accomplished by a) investigating the individual response patterns of AUDIT and identify latent groups using latent class analyses (LCA). Next, b) the associations between the classes identified in the best fitting LCA-model and sociodemographic factors will be analysed and presented. The sociodemographic factors included are age, gender, educational level, employment level, occupational level and income.

## Methods

### Design and setting

The present study has a cross-sectional design, and is part of the ongoing Norwegian national WIRUS project (“Workplace Interventions preventing Risky Use of alcohol and Sick leave”). Data for the current study was obtained from the alcohol screening component of WIRUS project. More details and other results from the WIRUS can be found elsewhere [19–24].

### Participants and data collection

In the WIRUS screening study, 20 large companies in Norway were recruited. These private (*n* = 8) and public sector (*n* = 12) companies employed approximately 18,000 persons. Individual-level criteria for being included in the screening study were: a) aged 16–72, b) status as an employee (i.e., salaried person) (c) employed in a company served by one of the participating Occupational Health Service units, regardless of work division or geographical region, d) basic understanding of the Norwegian language, and e) provided written informed consent to participate. Included companies provided email addresses for all their employees. Employees (*n* = 18,000) were invited to participate by receiving a web-based questionnaire. A total of 5136 employees responded on the questionnaire (28.5%), while 4311 (24.0%) responded on relevant items for this study and thus constitute the final study sample.

### AUDIT

A translated Norwegian full version of the AUDIT was used in the present study, consisting of 10 items measuring different aspects of alcohol habits during the last 12 months and potential negative consequences of these alcohol habits [5, 6]. A recent confirmatory factor analysis of AUDIT based on the WIRUS-study concluded that AUDIT consists of one factor, and that there were no indications of differences in the factor structure or metric across gender [22]. All 10 items were used as individual indicators in the latent class analysis.

### Covariates

Gender, age and educational level was recorded based on self-report information. Age was divided into five groups for the purposes of this study: 18–29, 30–39, 40–49, 50–59, and ≥ 60 years. Educational level was recorded as a four-level variable, discriminating between primary/lower secondary, upper secondary, university/college ≤4 years, and university/college > 4 years.

#### Full-time employment

The participants could indicate the current percentage of employment. In the present study the variable full-time employment was divided into those with less than full-time employment (< 100%) and those with full-time (37.5 h a week) or more.

#### Occupational level

The participants could indicate their occupational level according to four categories: 1) Employee, 2) Middle management, 3) Upper management and 4) Other (e.g. substitute, apprentice). Four percent indicated ‘other’ as occupational level and were excluded from this study due to the lack of specificity of the content. The three former categories were retained and used in the present study.

#### Income

The yearly household income was asked for in an open-ended question. In the present study, income was used as quintiles to mitigate potential problems regarding distribution and misclassification. A little over 5 % (*N* = 228) had missing information about yearly family income.

### Statistical analysis

#### Identification of number of classes and description of retained classes

**Fig. 1:**
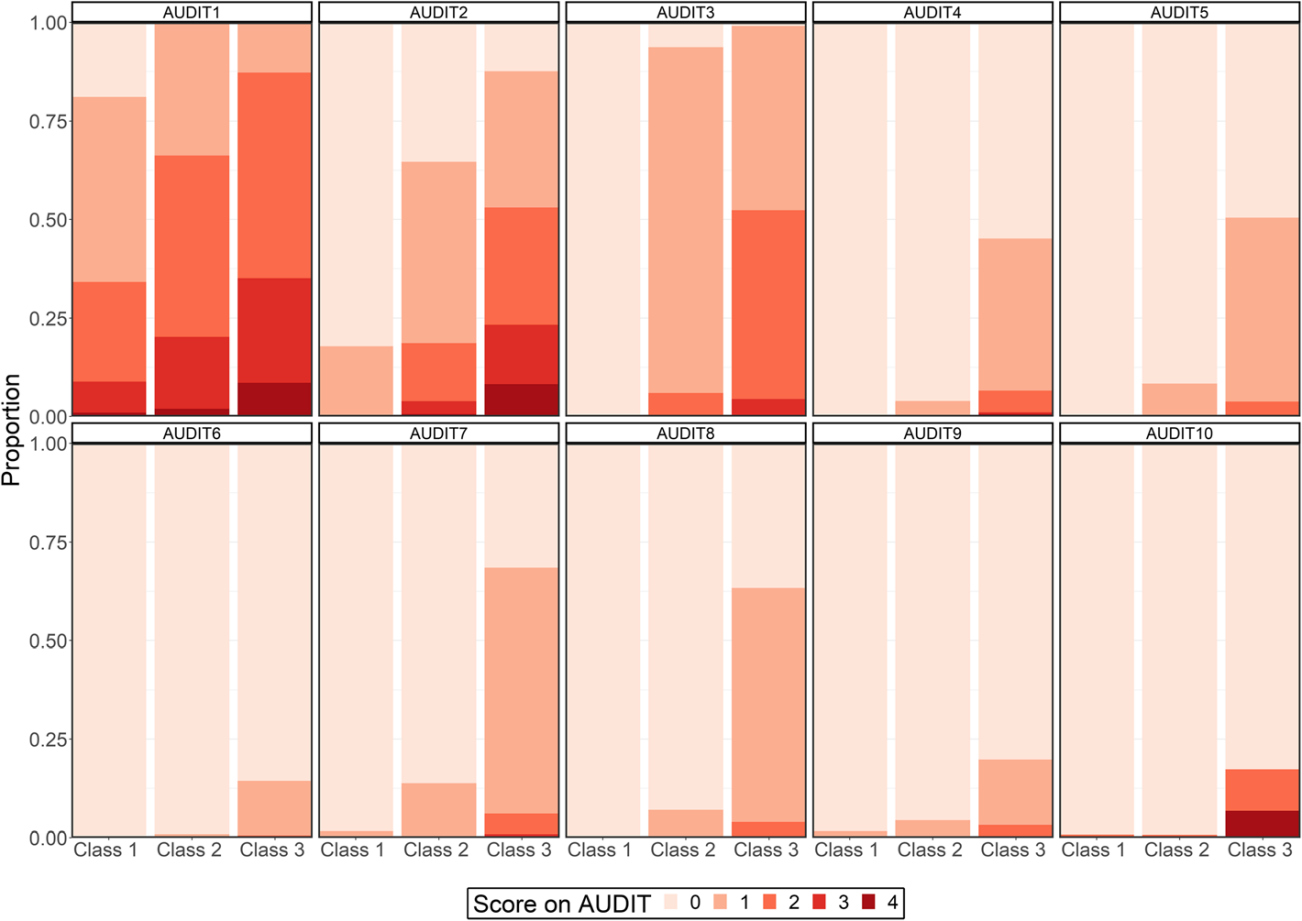
Response probability on AUDIT items across retained classes

Latent class analysis (LCA) was used to identify subsets of participants who shared a similar response pattern on the ten items of AUDIT. LCA can be understood as a person-centred approach that estimates the number of latent classes that can be discerned based on the total pool of individual response patterns. By using latent class analysis we tried to identify groups based on their pattern of responses across all 10 AUDIT items rather than merely using previously established cut-points or simple combinations of summed scores. The LCA-approach can be useful for exploring patterns in the data beyond a priori established categories, and may reveal sub-groups which are not directly observable by the indicators [25]. The approach is probabilistic rather than deterministic, and when deciding on the number classes to retain statistic criteria, parsimony and meaningfulness of the classes should be collectively considered [25]. The following statistical criteria were used to decide on the number of classes to retain: Consistent Akaike information criterion (AIC), Bayesian information criterion (BIC) and adjusted BIC (aBIC) [26], where lower values indicate better model fit (Table 1). Also, we used entropy to assess the quality of classification (ranging from 0 to 1 with higher values indicating better discrimination between classes), as well as the likelihood-ratio between the different models. The LCA was done iteratively, beginning with one class (i.e. similar response patterns across all participants), and increasing the number of classes up to 5. For each iteration, likelihood-ratio tests were employed to determine whether a model with one more class performs better than k-1 classes (Vuong-Lo-Mendell-Rubin likelihood ratio test and Lo-Mendell-Rubin adjusted likelihood-ratio test). After identification of the number of classes that best fitted the data, the response patterns across AUDIT items was presented for each of the classes (Fig. 1). As the AUDIT items differ in number of response options, the response patterns were also presented as binary variables differentiating between scoring 0 and more than zero (i.e. endorsing an item) of the probability of endorsing that particular item (Table 2).

**Table 1:** Comparison of model fit from 1 to 5 classes

Model	log-likelihood	resid. df	BIC	aBIC	cAIC	Likelihood-ratio	Entropy	VLMR-LRT	LMR-A-LRT
1 class	−24,274.68	4278	48,825.54	48,720.68	48,858.54	10,949.552	–	–	–
2 classes	−21,761.46	4244	44,083.64	43,870.74	44,150.64	5923.110	0.769	***p*** **< .0001**	***p*** **< .0001**
3 classes	−21,050.71	4210	42,946.68	42,625.74	43,047.68	4501.606	0.825	***p*** **< .0001**	***p*** **< .0001**
4 classes	−20,855.28	4176	42,840.36	42,411.39	42,975.36	4110.743	0.755	*p* = .7537	p = .7537
5 classes	−20,681.74	4142	42,777.83	42,240.82	42,946.83	3763.674	0.758	*p* = .5294	*p* = .5306

Bold indicates statistically significant differences

*BIC* Bayesian Information Criteria

*AIC* Aikaike Information Criteria

*VLMR-LRT* Vuong-Lo-Mendell-Rubin likelihood-ratio test for k-1 versus k classes

*LMR-A-LRT* Lo-Mendell-Rubin adjusted likelihood-ratio test for k-1 versus k classes

**Table 2:** Probability of endorsing (scoring more than 0) on AUDIT items across retained classes

	Class 1 (38.2%)	Class 2 (47.2%)	Class 3 (14.6%)
Item 1			
‘How often do you have a drink containing alcohol’	81.2%	99.8%	100.0%
*Frequency (Never 1x or less a month; 2-4x a month; 2-3x a week; 4x or more a week)*			
Item 2			
‘How many drinks containing alcohol do you have on a typical day when you are drinking’	17.9%	64.7%	87.6%
*Quantity (1–2 drinks; 3–4 drinks; 5–6 drinks; 7–9 drinks; 10 or more drinks)*			
Item 3			
‘How often do you have six or more drinks on one occasion’	0.1%	93.7%	99.1%
*Heavy episodic drinking (Never; Seldom; Monthly; Weekly; Daily)*			
Item 4			
‘How often have you found that you were not able to stop drinking’	0.1%	4.0%	45.2%
*Not able to stop drinking (Never; Seldom; Monthly; Weekly; Daily)*			
Item 5			
‘How often have you failed to do what was normally expected of you because of drinking’	0.3%	8.4%	50.5%
*Failed to do what is expected (Never; Seldom; Monthly; Weekly; Daily)*			
Item 6			
‘How often have needed a first drink in the morning […] after a heavy drinking session’	0.1%	0.9%	14.4%
*(Never; Seldom; Monthly; Weekly; Daily)*			
*Eye-opener*			
Item 7			
‘How often have you had a feeling of guilt or remorse after drinking’	1.6%	13.8%	68.5%
*Guilt (Never; Seldom; Monthly; Weekly; Daily)*			
Item 8			
‘How often have you been unable to remember what happened […] because of you drinking’	0.1%	7.0%	63.3%
*Memory loss (Never; Seldom; Monthly; Weekly; Daily)*			
Item 9			
‘Have you or someone else been injured because of you drinking’	1.6%	4.4%	19.8%
*Injury (No; Yes, but not during the last year; Yes, during the last year)*			
Item 10			
‘Have others been concerned about your drinking or suggested you cut down’	0.8%	0.7%	17.3%
*Concern/Cut-down (No; Yes, but not during the last year; Yes, during the last year)*			

Items 1–8 have five levels yielding scores between 0 and 4

Items 9 and 10 have three levels yielding the score 0, 2 or 4

Class 1: ‘Low-level consumption, no negative consequences’

Class 2: ‘Moderate level consumption, almost no negative consequences’

Class 3: ‘Higher-level consumption, prone to negative consequences’

#### Exploratory post-hoc latent class analyses

In an effort to explore the robustness of our identified classes we also performed two sets of exploratory post-hoc latent class analyses in two different sub-samples. The first sub-sample consisted of those scoring 8 or more on AUDIT which is the conventional cut-off for differentiating between low risk drinking and risky drinking [5]. The second sub-set consisted of those most likely to belong to class 3 described below. In each sub-sample we performed LCA to see if more than one class could be identified.

#### Association with covariates

**Fig. 2:**
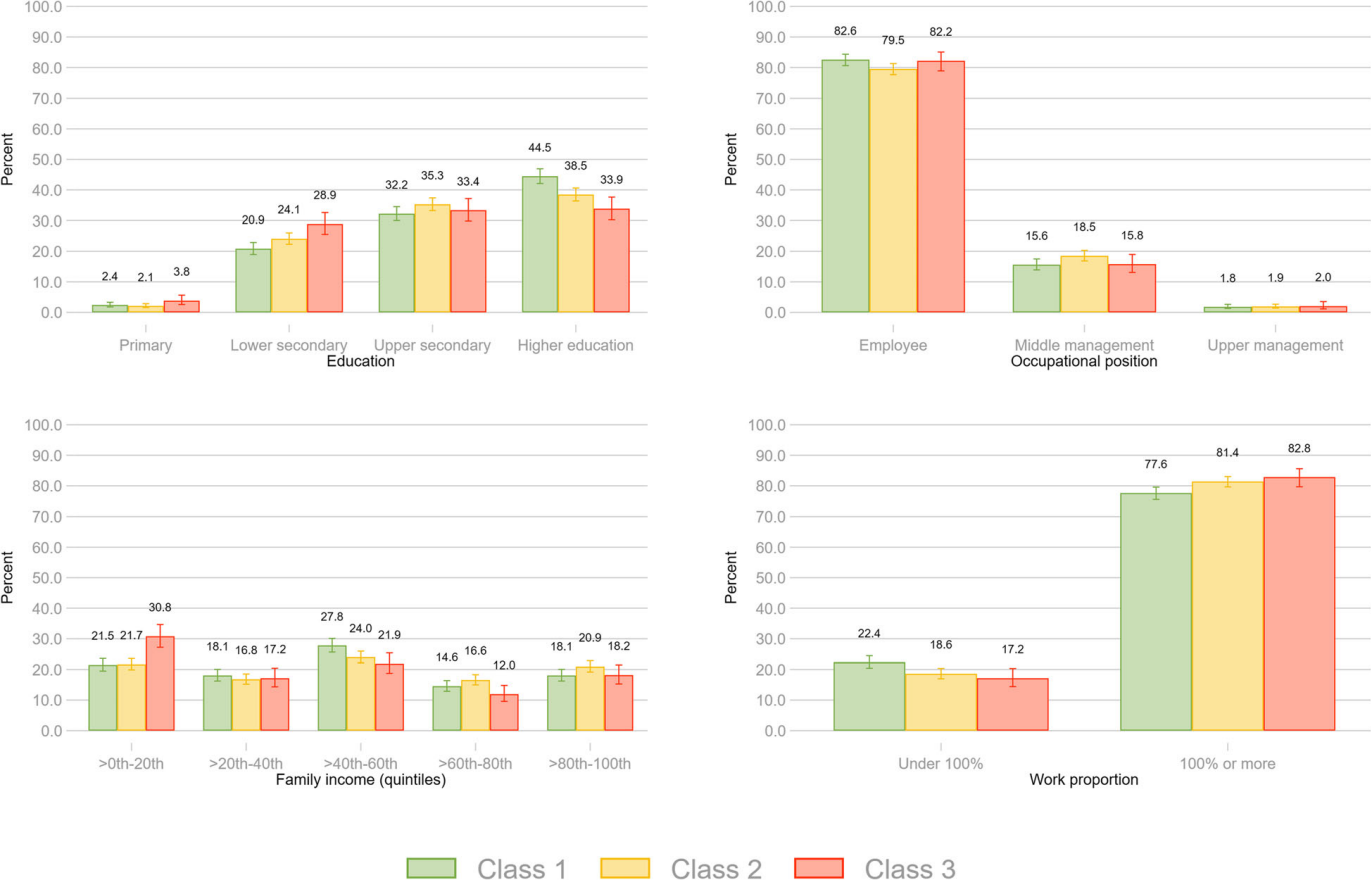
Distribution of indicators of socioeconomic status across classes. Crude proportions based on most probable class belongingness. Error bars denote 95% confidence intervals

The association between covariates and the latent classes was done using two different steps. First, the association between most likely class membership and covariates was estimated using traditional multiple logistic regression models with most probable manifest class membership as the dependent variable (Fig. 2). Second, the association was estimated using the 3-step approach recommended by Vermunt [27] and available in Mplus as the ‘R3STEP’-procedure [28]. The latter procedure yields estimates from multiple logistic regression models while also taking into account the probabilistic nature of the LCA approach (Table 3). The associations were estimated for each covariate separately. Only the odds ratios with *p*-values from the R3STEP-procedure is presented, as both approaches yielded very similar results. R [29] and the poLCA package [30] was used for the initial LCA, while inclusion of covariates using the ‘R3STEP’-procedure as well as likelihood-ratio tests was done in the Mplus statistical package version 8 [31].

**Table 3:** Comparison of class belongingness across covariates

	Age (S.E.; *p*-value)	Gender (S.E.; *p*-value)	Education (S.E.; *p*-value)	Occupational level^a^ (S.E.; *p*-value)	Full-time employment (S.E.; *p*-value)	Income (quintiles)^b^ (S.E.; *p*-value)
Unadjusted						
Class 2 (ref) vs 1	**1.38** **(.044;** ***p*** **< 0.001)**	**1.71** **(.139;** ***p*** **< 0.001)**	**1.13** **(.049;** ***p*** **= .009)**	**0.84** **(.070;** ***p*** **= .024)**	**0.79** **(.070;** ***p*** **= .002)**	**0.95** **(.025;** ***p*** **= .031)**
Class 3 (ref) vs 1	**1.65** **(.080;** ***p*** **< 0.001)**	**3.30** **(.357;** ***p*** **< 0.001)**	**1.38** **(.083;** ***p*** **< 0.001)**	0.98 (.121; *p* = .849**)**	**0.70** **(.094; p = .002)**	**1.13** **(.045;** ***p*** **= .005)**
Class 3 (ref) vs 2	**1.19** **(.059;** ***p*** **= .001)**	**1.94** **(.210;** ***p*** **< 0.001)**	**1.22** **(.075;** ***p*** **= .003)**	1.16 (.146; *p* = .273**)**	0.89 (.126; *p* = .397)	**1.19** **(.050;** ***p*** **< 0.001)**
Fully adjusted^c^						
Class 2 (ref) vs 1	**1.51** **(.054;** ***p*** **< 0.001)**	**1.87** **(.169;** ***p*** **< 0.001)**	**1.28** **(.064;** ***p*** **< 0.001)**	**0.83** **(.075;** ***p*** **= .022)**	0.86 (.088; *p* = .102)	**0.90** **(.026;** ***p*** **< 0.001)**
Class 3 (ref) vs 1	**1.77** **(.096;** ***p*** **< 0.001)**	**3.45** **(.425,** ***p*** **< 0.001)**	**1.44** **(.103,** ***p*** **< 0.001)**	0.87 (.122; *p* = .278)	**0.76** **(.123;** ***p*** **= .046)**	1.06 (.051; *p* = .227)
Class 3 (ref) vs 2	**1.18** **(.065;** ***p*** **= .006)**	**1.85** **(.223;** ***p*** **< 0.001)**	1.13 (.081; *p* = .117)	1.05 (.148; *p* = .749)	0.88 (.148; *p* = .420)	**1.18** **(.059; p = .002)**

Bold indicates statistical significance at ***p*** **<** .05

S.E.: standard error

Ref: Reference (base) class for comparison of two classes (multinomial logistic regression)

Reference categories for covariates: age (18–29 yrs), gender (male), education (primary/lower seconday), occupational level (employee), full-time employment (less than full-time employment), income (1st quintile)

^a^*N* = 173 deleted observations due to missing specific information regarding occupational level

^b^*N* = 228 deleted observations due to missing information regarding family income

^c^Model include age, gender, education, occupational level, full-time employment, income and company in same multinomial logistic regression (*N* = 3925)

### Ethical considerations

The study was approved by the Regional Committee for Medical and Health Research in Norway (REK) (approval no. 2014/647). Participants provided written informed consent upon participation.

## Results

Among the eligible participants, 67% were female, and the mean age for the eligible participants was 45.0 (standard deviation 11.6) years. Only one in forty reported primary/lower secondary education, 24% reported upper secondary, 34% reported ≤4 years of university/college education, while 40% reported more than four years of university/college education. Almost 80% reported full-time employment or more, the most common occupational level was employee (81%), and 17% were middle management. The median reported household income was 918,000 Norwegian Kroner (NOK; approximately 91,890€ September 2019), with an interquartile range of 530,000 NOK (53,050€).

### Number of classes

Compared with a 1-class solution, there was a substantial decrease in the values related to model fit for a 2-class solution (Table 1). There was a further substantial decrease when moving from a 2- to a 3-class solution, while the entropy increased from 0.769 to 0.825. When estimating more than 3 classes, however, the decrease in values related to model fit was comparatively small, and a drop in entropy was also observed. The likelihood-ratio tests also indicated there was no statistical improvement of the model by allowing for more than three classes. Based on this, and after visual inspection of the meaningfulness of classes 2, 3 and 4, it was decided to retain the 3-class solution.

### Characteristics of retained classes

For class 1 there was a relatively high probability (81.2%) for endorsing (scoring more than 0 points) on item 1, but a rather low probability (17.9%) for endorsing item 2 (for more details see Fig. 1 and Table 2). For the rest of the items, the probability of endorsing was negligible. The mean sum score for class 1 was 1.6 (median 1, interquartile range 1–2).

For class 2 there was very high probability for endorsing item 1 (99.8%) and 3 (93.7%), and relatively high probability of endorsing item 2 (64.7%). For the rest of the items the probability of endorsing ranged from negligible (item 6 and 10) to relatively low (items 4, 5, 7, 8 and 9). The mean sum score for class 2 was 4.3 (median 4, interquartile range 3–5).

All the members of class 3 scored endorsed item 1 (100%) and had a very high probability of endorsing item 2 (87.6%) and item 3 (99.1%). Furthermore, there was over 50% probability endorsing item 5, 7 and 8, while the probability for endorsing item 4 was 45.2%. The probability for endorsing the rest of the items (items 6, 9 and 10) was moderate ranging between 14.4 and 19.8%. The mean sum score for class 3 was 9.4 (median 9, interquartile range 7–10).

Based on the response patterns of the classes, they were characterised as follows: ‘Class 1: Low-level consumption, no negative consequences’ (38.2%), ‘Class 2: Moderate level consumption, almost no negative consequences (47.2%)’, and ‘Class 3: Higher-level consumption, prone to negative consequences (14.6%)’.

#### Exploratory post-hoc latent class analyses

The post-hoc analysis of the sub-group defined by those scoring 8 or above on the AUDIT-sum score (*n* = 486) did not indicate more than one class as the fit indices were only marginally improved with increasing number of classes (data not shown). Furthermore, the likelihood-ratio tests did not support more than one class (both *p*-values > 0.05 for 2 classes versus 1 class). Additional sub-group analysis was performed by establishing a sub-group for those most with most likely membership to ‘Class 3: Higher-level consumption, prone to negative consequences’ (*n* = 629). Also in this sub-group there was little support for more than 1 class based on the fit indices (data not shown), which was supported by the likelihood-ratio tests (both *p*-values for > 0.05 for 2 classes vs 1 class). The post-hoc latent class analyses did not support additional classes beyond those identified in the main analysis.

### Class belongingness and covariates

When comparing class 1 with class 2, there was an increased odds of belonging to class 1 with increasing age (odds ratio (OR) 1.38), being female (OR 1.71), having higher education (OR 1.13), while there was a decreased odds of having a higher occupational level (i.e. managerial role; OR 0.84), full-time employment (OR 0.79) and higher income (OR 0.95; Table 3). In the adjusted model the same pattern of associations were observed but the association with full-time employment was no longer statistically significant (see Fig. 2 for crude proportion across indicators of socioeconomic status and Table 3).

When comparing class 1 with class 3, there was an increased odds of belonging to class 1 with increasing age (OR 1.65), being female (OR 3.30), having higher education (OR 1.38) and higher income (OR 1.13), while there was a decreased odds of having a full-time employment (OR 0.70). There was no difference between class 1 and 3 with regards to occupational level. In the adjusted model a similar pattern was observed, but the difference in income was no longer statistically significant (Table 3).

When comparing class 2 with class 3, there was an increased odds of belonging to class 2 with increasing age (OR 1.19), being female (OR 1.94), having higher education (OR 1.22) and higher income (OR 1.19). There was no difference between class 2 and 3 with regards to occupational level or full-time employment. In the adjusted model, the associations were similarly patterned, but education was no longer significant.

Overall, there were only small differences between the crude association estimates and the adjusted ones, and four associations were rendered non-significant (Table 3).

Upon investigating the distribution of socioeconomic status across the three classes, some notable differences are worth mentioning (Fig. 2): Those belonging to class 3 report a higher proportion of only primary education (3.8%) and a lower proportion of higher education (33.9%) compared to the two other classes (Fig. 2). On the other hand, class 1 reported a substantially higher proportion of higher education (44.5%) compared to the remainder. Furthermore, over 30% of those belonging to class 3 reported a family income in the lowest quintile, compared to around 22% in the other classes.

## Discussion

### Main findings

In the present study, we found support for 3 substantive classes based on the response patterns on AUDIT in a cohort of Norwegian workers. Class 1 could adequately be described in terms of low-level alcohol consumption both with regards to frequency and intensity/‘binge drinking’, as well as reporting a very low probability of negative consequences (as measured by items 4–10 on AUDIT) related to their alcohol consumption. Class 2 was characterised by a higher level of consumption both in terms of frequency and intensity, but despite this, class 2 also had a relatively low probability of reporting any negative consequences related to their alcohol consumption. The last class, however, was characterised by even higher levels of frequency and intensity of consumption, as well as a rather high probability of reporting negative consequences of their consumption. Exploratory post-hoc analyses did not support additional classes in sub-group analyses.

In relation to the included covariates, important differences were observed across classes. For age, gender and education, class 1 was characterised by older age, a higher proportion of females and higher educational attainment, and class 3 by younger age, more males and lower educational attainment. Class 2 fell somewhere in between these two classes with regards to these factors. For income, class 2 was characterised by higher income compared to the other classes, and class 3 were characterised by the lowest level of income. Comparing work-related factors, the differences were less overarching, and occupational level only differed between class 1 and class 2, where the latter class was characterised by higher occupational level. For full-time employment, class 1 was more likely to report less 100% occupation compared to the remaining classes. In the adjusted models, four of the associations were rendered non-significant, but only small changes to the point estimates were observed indicating little confounding. No other differences were observed between classes for the included covariates.

### Interpretation of findings

The three different classes we identified makes intuitively sense with regards to the relationship between alcohol consumption patterns (AUDIT items 1–3) and self-reported alcohol-related consequences (AUDIT items 4–10). Our results also yields indirect support for the conventional cut-point of 8 for alcohol-related problems as the central tendency scores (mean and median) of the class described as ‘higher-level consumption, prone to negative consequences’ (class 3) was close to the suggested cut-point while the other classes central tendency scores were well below [5]. The general distribution of age, gender and education across classes is in line with previous findings from the WIRUS-study where cut-points and the sum score of AUDIT was used [21]. With regards to findings from other studies, we found that income and occupational level is independently differentially associated with the retained classes in mostly expected ways [3, 12, 32]. However, we also found that those with lower occupational level (compared to class 2), less income (compared to class 2) and less than 100% employment (compared to class 2 and 3) are more likely to belong to the low risk class (class 1), even though they are more likely to report higher education in this class (compared to class 2 and 3). This could be due to gender differences in the association between education and occupational for women) [33], and not a reflection of actual disparities between education and the other socioeconomic indicators in relation to alcohol. This notion was, however, not reflected in our findings as there was only small changes from unadjusted to adjusted estimates, despite the tendency for socioeconomic factors to cluster and co-vary. Residual confounding is however always an issue and it is possible that inclusion of other unmeasured covariates would have yielded greater evidence for confounding.

Some previous studies have adjusted for drinking patterns when investigating the association between SES and alcohol-related consequences [12, 15]. Our findings indicate that individual drinking patterns is intrinsically related to self-reported negative consequences, as the only identified class (class 3) with a high probability of reporting negative consequences of their alcohol habits also was the class characterised by substantially higher volume of consumption (quantity and frequency, item 1 and 2) as well as frequency of binge drinking (item 3). This suggests that the level of alcohol consumption and consumption patterns, and the consequences cannot be understood separately from each other. Class 3 also differed on key demographic and socioeconomic indicators from the other classes in meaningful ways – characterised by lower age, more males, lower education and lower income. Taken together, the clear relationship between alcohol consumption patterns (AUDIT items 1–3), self-reported alcohol-related consequences (AUDIT items 4–10) and socioeconomic status suggest that adjusting for alcohol consumption when investigating the association between socioeconomic status and alcohol harm may be futile due to their interconnectedness. Rather, alcohol habits and alcohol-related factors should be seen in conjunction in further research and efforts should be made to identify meaningful patterns and assess status and objective measures of alcohol harm.

### Strengths and limitations

The present study is characterised by several strengths. First, the large study size enabled identification of different sub-groups as determined by their alcohol consumption pattern and related consequences using latent class analysis. Relatedly, the study uses a compound measure of alcohol, which incorporates both aspects related to alcohol consumption (i.e. quantity, frequency and binge drinking), and self-reported negative consequences of the consumption (i.e. injury, memory loss, and reduced functioning). Second, our study included several measures of socioeconomic status such as educational attainment and income. This enabled a more detailed investigation into differential association between different classes and aspects of socioeconomic status. Several limitations are worth mentioning. First, given the low participation rate, our findings should be interpreted with caution, as they may not be representative of the whole invited sample. Due to data protection regulations, we are not able to compare non-participants and participants directly, but comparisons between the invited sample and the participants finds that gender composition among the participants are similar to the invited sample (*p* = 0.172). Those participating were, however, somewhat older compared to the invited sample (*p* < 0.001; 68.1% aged 40 or above among the participants versus 63.7% in the invited sample). The participants were recruited from a wide range of private and public enterprises. However, previous studies have reported that the WIRUS-sample is characterized by an overrepresentation of older, highly educated, and female employees compared to the entire Norwegian workforce [19, 21]. On the other hand, the WIRUS-sample can be considered more representative when it comes to the composition of gender and educational attainment among public and state sector employees. These considerations may limit the generalisability and external validity of the findings from the present study. Also, our findings are not necessarily generalisable to other populations. Most of our participants had an employment size of 100% or more, and the lack of variation thereof limited our ability to analyse differences with respect to employment size. Ideally, the distribution of employment size should be wider in order to investigate this indicator more fully. Furthermore, we were only able to discriminate between three broad levels of occupation. Ideally, a higher degree of differentiation would be preferable to shed more light on the role of education. Third, the WIRUS-study does not include questions regarding other life-style or health factors, such as smoking, diet, physical activity, general or mental health. Inclusion of such factors would have yielded more information regarding the relationship between the identified classes and measure of socioeconomic status, such as for instance abstention from drinking due to chronic health conditions or former alcohol-related problems. Fourth, despite the inclusion of several measures of socioeconomic status, even more measures would increase the value of our findings. This includes for instance area- or neighbourhood-based deprivation, home/car ownership as socioeconomic status is a complex phenomenon with many facets. Fifth, we were not able to identify a class defined by consistently very high scores across AUDIT-items. This would have been interesting based on previous studies which have highlighted high-risk alcohol groups (see for instance Lewer and colleagues [8]) as particularly interesting for understanding the alcohol-harm paradox. The range of AUDIT scores are limited (0–26), and less than 1% of participants scored more than 16 in total, and this is probably partly due to being recruited from a working population.

### Implications

The findings from the latent class analyses identified a rather large class (≈15%) with both higher levels of alcohol consumption and a proneness to negative consequences related to their drinking. We were not able to further differentiate this group, and despite being more likely to report negative consequences must be regarded as within the lower end of the risk spectrum as described by Babor and colleagues [5]. That would translate into simple advice as the recommended intervention in a public health perspective [21]. Our findings further highlight a need for differentiation of various aspects of alcohol-related harm and behaviour, and demonstrate a differential association of individual factors often used to gauge SES. Specifically, our findings indicate that being young, male, having low educational attainment and low income were associated with particular exposure to both high levels of alcohol consumption and alcohol-related harm. Such knowledge may have practical implications for alcohol-preventive efforts within the frame of workplace interventions. For instance, companies that to a large extent employ individuals associated with these sociodemographic characteristics, should make alcohol-preventive efforts an overall priority both in terms of general alcohol education, but also more targeted approaches for those that report both higher levels of alcohol consumption and a proneness to negative consequences related to their alcohol habits.

### Future direction: the alcohol harm paradox

As mentioned in the introduction, both ‘differential exposure’ and ‘differential vulnerability’ is relevant when trying to understand mechanisms underlying social inequalities in health. Regarding alcohol harm and differential vulnerability, several aspects may be relevant. First, it is plausible that other life-style factors, such as smoking, unhealthy diet and less physical activity, increases the vulnerability for exposure to alcohol. A recent study concluded for instance that combinations of lifestyle factors such as smoking, excessive alcohol consumption, poor diet and low physical activity is associated with an excess risk for poor health in socioeconomically deprived populations [1]. Despite such findings, Katikreddi and colleagues (2017) reported that smoking and obesity could not help explain the increased alcohol-attributable harm observed among lower versus higher socioeconomic groups [15]. Studies informed by twin studies also indicate that alcohol habits are associated with socioeconomic indicators that cannot be explained by shared exposures or a range of potential confounders [18]. Second, other factors such as chronic stress [34], stressful life-events [35, 36], stigma [37], mental health problems [38] and poor health in general [39] may put individuals in lower socioeconomic status groups at a higher risk for alcohol harm despite similar consumption patterns. Third, a more limited access to high-level quality health care [40, 41] or less efficient application of available health information (‘health literacy’) [42, 43] may also result in excess exacerbation of symptoms or conditions attributable to alcohol in groups with lower socioeconomic status compared to groups higher on the socioeconomic ladder. The abovementioned vulnerability-factors may modify the relationship between alcohol exposure and alcohol-related harm in complex ways. We are not aware of studies which have specifically investigated this and future research need to further address differential exposure and differential vulnerability as a potential mechanism. In that respect an evidence review, Roche and colleagues highlighted some of the challenges with respect to giving an overview of social inequality in alcohol-related health [2]:

1. The use of different measures of socioeconomic status as well as alcohol use across studies
2. Different determinants can interact with each other in a multitude of ways
3. The different components of socioeconomic status can act as mediators for each other
4. Disadvantaged groups may be encumbered by several risk factors, which in turn can interact and modify each other

Navigating these abovementioned factors in future research will provide further knowledge about the true relationship between alcohol consumption patterns, alcohol harm and socioeconomic status.

## Conclusions

We found evidence for three latent classes based on the participants’ response pattern on AUDIT. The class reporting the lowest levels of alcohol consumption, also reported the lowest level of negative alcohol-related consequences. The class with the highest levels of alcohol consumption, also reported the highest level of negative consequences. This highlight the interconnectedness of alcohol consumption and alcohol-related consequences, and also suggest that efforts to disentangle them is very challenging. Our finding that the classes identified were systematically differentially associated with the included measures of socioeconomic status yields further insights into to intricate relationship between various socioeconomic factors, alcohol use patterns and related negative consequences. Our findings indicate that work places characterized by younger, male employees with lower educational attainment and lower wages should incorporate both general alcohol education and targeted alcohol interventions when necessary in their occupational health efforts.

## Data Availability

The dataset used and analysed during the current study is available from the corresponding author on reasonable request.

## Abbreviations

AUDIT: The Alcohol Use Disorders Identification Test
CI: Confidence interval
LCA: Latent class analysis
OR: Odds ratio
REF: Reference category
REK: Regional Committees for Medical and Health Research in Norway
S.E.: Standard error
WHO: World Health Organization
WIRUS: Workplace Interventions preventing Risky Use of alcohol and Sick leave

## References

[1] Foster HME (2018). The effect of socioeconomic deprivation on the association between an extended measurement of unhealthy lifestyle factors and health outcomes: a prospective analysis of the UK biobank cohort. Lancet Public Health.

[2] Roche A (2015). Evidence review: the social determinants of inequities in alcohol consumption and alcohol-related health outcomes.

[3] Beard E (2016). Deconstructing the alcohol harm paradox: a population based survey of adults in England. PLoS One.

[4] Bloomfield K (2006). Social inequalities in alcohol consumption and alcohol-related problems in the study countries of the EU concerted action 'Gender, culture and alcohol problems: a multi-national Study'. Alcohol Alcohol Suppl.

[5] Babor T (2001). AUDIT. The Alcohol Use Disorders Identification Test: Guidelines for Use in Primary Care.

[6] Saunders JB (1993). Development of the alcohol use disorders identification test (AUDIT): WHO collaborative project on early detection of persons with harmful alcohol consumption--II. Addiction.

[7] Hall W (2017). Socioeconomic status and susceptibility to alcohol-related harm. Lancet Public Health.

[8] Lewer D (2016). Unravelling the alcohol harm paradox: a population-based study of social gradients across very heavy drinking thresholds. BMC Public Health.

[9] Diderichsen F, Evans T, Whitehead M (2001). The social basis of disparities in health, in Challenging inequities in health. From ethics to action, T. Evans, et al., Editors.

[10] Probst C (2014). Socioeconomic differences in alcohol-attributable mortality compared with all-cause mortality: a systematic review and meta-analysis. Int J Epidemiol.

[11] Beard E (2019). Associations between socio-economic factors and alcohol consumption: a population survey of adults in England. PLoS One.

[12] Huckle T, You RQ, Casswell S (2010). Socio-economic status predicts drinking patterns but not alcohol-related consequences independently. Addiction.

[13] Giskes K (2011). Individual and household-level socioeconomic position is associated with harmful alcohol consumption behaviours among adults. Aust N Z J Public Health.

[14] Pena S (2017). Socioeconomic inequalities in alcohol consumption in Chile and Finland. Drug Alcohol Depend.

[15] Katikireddi SV (2017). Socioeconomic status as an effect modifier of alcohol consumption and harm: analysis of linked cohort data. Lancet Public Health.

[16] Jones L (2015). Relationship between alcohol-attributable disease and socioeconomic status, and the role of alcohol consumption in this relationship: a systematic review and meta-analysis. BMC Public Health.

[17] Bellis MA (2016). The alcohol harm paradox: using a national survey to explore how alcohol may disproportionately impact health in deprived individuals. BMC Public Health.

[18] Bockerman P, Hyytinen A, Maczulskij T (2017). Alcohol consumption and long-term labor market outcomes. Health Econ.

[19] Aas RW (2017). The influence of alcohol consumption on sickness presenteeism and impaired daily activities. The WIRUS screening study. PLOS ONE.

[20] Nordaune K (2017). Who initiates and organises situations for work-related alcohol use? The WIRUS culture study. Scand J Public Health.

[21] Thørrisen MM, Skogen JC, Aas RW (2018). The associations between employees' risky drinking and sociodemographics, and implications for intervention needs. BMC Public Health.

[22] Skogen JC, Thørrisen MM, Olsen E, Hesse M, Aas RW. Evidence for essential unidimensionality of AUDIT and measurement invariance across gender, age and education. Results from the WIRUS study. Drug and alcohol dependence. 2019;202:87-92. 10.1016/j.drugalcdep.2019.06.002 31325821

[23] Thørrisen MM (2019). Association between alcohol consumption and impaired work performance (presenteeism): a systematic review. BMJ Open.

[24] Thørrisen MM (2019). Current practices and perceived implementation barriers for working with alcohol prevention in occupational health services: the WIRUS OHS study. J Subst Abuse Treat Prev Policy.

[25] Porcu M, Giambona F (2017). Introduction to Latent Class Analysis With Applications.

[26] Dziak JJ (2019). Sensitivity and Specificity of Information Criteria.

[27] Vermunt JK (2010). Latent class modeling with covariates: two improved three-step approaches. Polit Anal.

[28] Asparouhov T, Muthén B. Auxiliary variables in mixture modeling: a 3-step approach using Mplus. Mplus Web Notes. 2013;15.

[29] R Core Team (2013). R: A language and environment for statistical computing.

[30] Linzer DA, Lewis JB (2011). poLCA: an R package for Polytomous variable latent class analysis. J Stat Softw.

[31] Muthén LK, Muthén B. Mplus User's Guide. Eighth Edition. Los Angeles CA: 1998-2017, Muthén & Muthén.

[32] Kinjo A (2018). Different socioeconomic backgrounds between hazardous drinking and heavy episodic drinking: prevalence by sociodemographic factors in a Japanese general sample. Drug Alcohol Depend.

[33] Skogen JC, Hensing G, Øverland S, Knudsen AK, Sivertsen B, Vahtera J, Haukenes I. The gender gap in accrued pension rights–an indicator of women’s accumulated disadvantage over the course of working life. The Hordaland Health Study (HUSK). Scand J Public Health. 2018;46(3):417–424. 10.1177/1403494817715845 28673123

[34] Baum A, Garofalo JP, Yali AM. Socioeconomic status and chronic stress: does stress account for SES effects on health?. Annals of the New York Academy of Sciences. 1999;896(1):131-44. 10.1111/j.1749-6632.1999.tb08111.x 10681894

[35] Keyes KM, Hatzenbuehler ML, Hasin DS (2011). Stressful life experiences, alcohol consumption, and alcohol use disorders: the epidemiologic evidence for four main types of stressors. Psychopharmacology.

[36] Lantz PM, House JS, Mero RP, Williams DR. Stress, life events, and socioeconomic disparities in health: results from the Americans' Changing Lives Study. Journal of health and social behavior. 2005;46(3):274-88. 10.1177/002214650504600305 16259149

[37] Room R. Stigma, social inequality and alcohol and drug use. Drug and alcohol review. 2005;24(2):143-55. 10.1080/09595230500102434 16076584

[38] Allen J, Balfour R, Bell R, Marmot M. Social determinants of mental health. International review of psychiatry. 2014;26(4):392-407. 10.3109/09540261.2014.928270 25137105

[39] Marmot M. Social determinants of health inequalities. The Lancet. 2005;365(9464):1099-1104. 10.1016/S0140-6736(05)71146-6 15781105

[40] Nunes BP, Thumé E, Tomasi E, Duro SMS, Facchini LA. Socioeconomic inequalities in the access to and quality of health care services. Revista de saude publica. 2014;48:968-76. 10.1590/S0034-8910.2014048005388 PMC4285826 26039400

[41] Dmitry T (2018). Local income inequality, individual socioeconomic status, and unmet healthcare needs in Ohio. USA..

[42] Frohlich KL (2016). Social inequalities in health information seeking among young adults in Montreal. Health Promot Int.

[43] Friis K (2016). Health Literacy Mediates the Relationship Between Educational Attainment and Health Behavior: A Danish Population-Based Study. J Health Commun.

